# Supplementary material for: Clinical characteristics and risk factors for rebleeding in uremic patients with obscure gastrointestinal bleeding undergoing deep enteroscopy: A multi-center retrospective study in Taiwan
Source: PLoS One. 2022 Nov 30;17(11):e0277434. doi: 10.1371/journal.pone.0277434 (PMC9710796; doi:10.1371/journal.pone.0277434)
Supplement: Attachment — Submitted filename: Reviewer_PLOS_N.docx [file pone.0277434.s002.docx]

Responses to Reviewers’ comments:

Reviewer #1: Authors proposed clinical characteristics and risk factors for rebleeding in uremic patients with obscure gastrointestinal bleeding undergoing deep enteroscopy. It would give another point of view for clinical daily practice. I have several questions as below.

1. In statistical analysis, continuous variables should be analyzed using the Mann–Whitney U test. Therefore, I suggest authors may recheck the P value for continuous variables in table 4 and 5.

A:

Thank you for your reminder. We rechecked the statistical analysis in detail and revised the manuscript in the “Materials and Methods” section and in Tables 4 and 5.

2. In table 4 and 5, authors presented the characteristics and "factors" between subgroups with/without rebleeding or with/without angiodysplsia. Moreover, authors should calculate the odds ratios for these risk factors which were determined through univariate and multivariate logistic regression analyses in another tables.

A:

Thank you for your comments. We reperformed the statistical analysis and added the odds ratios from univariate and multivariate analyses in Tables 4 and 5. In Table 4, two factors (valvular heart disease and use of anti-thrombotic agents—correlated with VHD in clinical use) were entered into the multivariate logistical regression model. In Table 5, only the P-value of “rebleeding” was significant on univariate analysis, which was then entered into the multivariate logistical regression model.

3. In table 4, I don't know the meaning (no data) about "location of bleeding" between subgroups with/without rebleeding.

A:

Thank you for your question. The location of bleeding refers to the location of bleeder. We revised Table 4 and performed statistical analysis further in different locations.

Reviewer #2: Appreciate the authors providing us about the risk factors for rebleeding in uremic patients with obscure GI bleeding

1. There is no clear mention about definition of "rebleeding" in the article. There is no detailed statements about lesions with rebleeding. What is the most common lesion type of rebleeding, angiodysplasia or ulcer?

A:

Thanks for your comment. The definition of rebleeding was described in “Materials and Methods” in the revised manuscript. However, not all patients with rebleeding underwent repeat deep enteroscopy; the actual etiology of rebleeding could thus not be clarified clearly. However, referring to the past literature and our indirect data (in the patients with different bleeding types, the rebleeding rate of angiodysplasia type vs. non-angiodysplasia type was 34% vs. 6.7%), the most common type of re-bleeding was likely to have been angiodysplasia. We put this issue into the “Discussion” section.

2. Eight patients with VHD and 4 of then with severe aortic stenosis and 4 patient were non-AS patients. Have you performed subgroup analysis for the relationship between OGIB and non-AS patients ? Many patients with VHD take antiplatelet or anticoagulation for prevention of thrombotic events. Have you analyze the effect of OGIB in anti-thrombotic agent for patient with VHD ? Your statistical analysis should manage the possible confounding factors of rebleeding.

A:

Given that only four patients had non-AS VHD, we did not further determine the relationship between OGIB and non-AS patients. As for the confounding effect of anti-thrombotic agents in patients with VHD, we adjusted for this factor using multivariate logistical regression analysis, and VHD still showed a statistically significant difference between the rebleeding and non-rebleeding groups (p=0.047).

3. No “Age” included in the clinical characteristics in table 1.

A: Thank you for pointing this out. The age has been included in the top row of Table 1 (marked in Red).

4. Table 1: What is the details of anti-thrombotic agents (antiplatelet? warfarin? DOAC?) Dose each subtype of anti-thrombotic agent have effect on OGIB?

A: In 25 patients who received antithrombotic agents (32.1%), 16 received aspirin and 9 received clopidogrel. We revised Table 1 accordingly. In our statistical analysis, the diffent types of anti-thrombotic agents showed no significalnt differences between the rebleeding and no rebleeding-groups (data no shown).

5. Table 3: Endoscopic intervention for what kinds of bleeder? Four patient received surgery or TAE and what is the cause of bleeding? Different etiology of bleeding may be one of the risk factor of rebleeding.

A:

Thank you for your comments. In our study, three patients underwent segmental bowel resection (two cases of jejunual angiodysplasia and one of ileal varices), and one patient with jejunal multiple angiodysplasia underwent transarterial embolization after failure of endoscopic intervention. This point was added to the “Results” section of the revised manuscript.

6. You did not mention the endoscopic appearance of angiodysplasia. There are different types of angiodysplasia (Yano –Yamamoto classification) and each had different rebleeding rate. You should analyze the rebleeding risk between different subtype of angiodysplasia.

A:

Thank you for your constructive comment. As per the Yano-Yamamoto classification, angiodysplasia was classified as type I (type Ia and Ib). Dieurofoy’s lesion was classified as type 2, AVM was classed as type 3, and unclassified lesion was classed as type 4. Therefore, our enrolled patients with angiodysplasia had type 1 vascular lesion and we did not determine the effect of different subtypes of angiodysplasia (type Ia and Ib) on rebleeding. However, whether the different types of vascular lesions in the small bowel can affect the rebleeding rate in uremic patients with OGIB needs further determination. We discussed this concern in the “Discussion” section.

7. There are multiple grammatical mistakes throughout the manuscript, I would recommend the authors revise the manuscript and consult English editing.

A:

Thanks for your positive comment. We have gotten the manuscript edited by a professional English language-editing service.
