# Supplementary material for: Clinical characteristics and risk factors for rebleeding in uremic patients with obscure gastrointestinal bleeding undergoing deep enteroscopy: A multi-center retrospective study in Taiwan
Source: PLoS One. 2022 Nov 30;17(11):e0277434. doi: 10.1371/journal.pone.0277434 (PMC9710796; doi:10.1371/journal.pone.0277434)
Supplement: Attachment — Submitted filename: Reviewer_POLS_20221024.docx [file pone.0277434.s003.docx]

Responses to Reviewers’ comments:

Reviewer #2: Appreciate the authors revising the manuscripts with efforts.

#1 Page 7 line 92-95

The sentence can be deleted “After being informed of the needs and potential complications of SBE, all patients signed an informed consent form. The requirement for patient consent for publication was waived because the patient information was anonymized and de-identified prior to analysis.”. You have repeated the same words in page 7, line 106-108.

A:

Thank you for your comment. We have revised the manuscript.

\# 2 Page 7 line 104

Why the definition of rebleeding is “ the evidence of recurrent overt gastrointestinal bleeding with recent negative EGD and colonoscopy and/or a drop in the Hb level…..” ? Why not enteroscopy (SBE)?

The purpose of manuscript as you mentioned was to “identify the clinical characteristics of uremic patients receiving SBE for OGIB and analyze the risk factors “

A:

Thank you for your comment. Among the 78 uremic patients with OGIB receiving SBE, not all patients with recurrent GI bleeding underwent repeat EGD, colonoscopy, or SBE. Therefore, we defined rebleeding as the evidence of recurrent overt gastrointestinal bleeding (melena or hematochezia) with recent negative EGD and colonoscopy and/or a drop in the hemoglobin level by >2 g/dl.

#3 Figure 1: I suggest that the author try to simplify the figure and remove redundant words. Otherwise this picture looks unnecessary.

A:

As suggested, we have simplified Figure 1 in the revised manuscript.

#4 Page 17 line 194-196 ??

“As lesions in the small intestine are similar to those in other gastrointestinal areas such as the stomach or large intestine, they can be diagnosed and treated by traditional EGD or colonoscopy. “

Does the author really express what you want to express correctly?

A:

Thank you for your kind comment. We have revised the content of this paragraph to express our meaning correctly.

#5 There are multiple grammatical mistakes throughout the manuscript, I strongly recommend the author revise the manuscript and consult English editing again.

A:

Thank you for pointing it out. I have further consulted an English editing service and revised this manuscript.
